# Association of nicotinamide-*N*-methyltransferase mRNA expression in human adipose tissue and the plasma concentration of its product, 1-methylnicotinamide, with insulin resistance

**DOI:** 10.1007/s00125-014-3490-7

**Published:** 2015-01-18

**Authors:** Aimo Kannt, Anja Pfenninger, Lenore Teichert, Anke Tönjes, Arne Dietrich, Michael R. Schön, Nora Klöting, Matthias Blüher

**Affiliations:** 1 Sanofi Research and Development, Industriepark Hoechst, H824, 65926 Frankfurt am Main, Germany; 2 Department of Medicine, University of Leipzig, Leipzig, Germany; 3 Department of Surgery, University of Leipzig, Leipzig, Germany; 4 Clinic of Visceral Surgery, Städtisches Klinikum Karlsruhe, Karlsruhe, Germany; 5 IFB Obesity Diseases, Junior Research Group Animal Models, University of Leipzig, Leipzig, Germany

**Keywords:** Adipose tissue, Insulin resistance, 1-Methylnicotinamide, Nicotinamide, Nicotinamide-*N*-methyltransferase, *NNMT*, Type 2 diabetes

## Abstract

**Aims/hypothesis:**

Nicotinamide-*N*-methyltransferase (NNMT) was recently shown to be upregulated in mouse models of insulin resistance and obesity. So far, it is unknown whether NNMT is regulated in human disease. We have explored the hypothesis that white adipose tissue (WAT) *NNMT* expression and plasma 1-methylnicotinamide (MNA) concentration are increased in human insulin resistance and type 2 diabetes.

**Methods:**

*NNMT* expression and plasma MNA were analysed in three groups of individuals: (1) 199 patients undergoing abdominal surgery; (2) 60 individuals on a 12-week exercise programme and (3) 55 patients on a two-step bariatric surgery programme.

**Results:**

Patients with manifest type 2 diabetes have a significantly (approximately twofold) higher *NNMT* expression both in omental and subcutaneous WAT compared with controls. Notably, plasma MNA correlated significantly with WAT *NNMT* expression in patients with type 2 diabetes (women, *r* = 0.59, *p* < 0.001; men, *r* = 0.61, *p* < 0.001) but not in healthy control individuals. In insulin-resistant individuals, there was an inverse correlation between insulin sensitivity and plasma MNA (*r* = 0.44, *p* = 0.01) or adipose tissue *NNMT* mRNA (*r* = 0.64, *p* < 0.001). The latter association was confirmed in a second cohort (*n* = 60, *r* = 0.78, *p* < 0.001). Interventions improving insulin sensitivity—exercise and bariatric surgery—were associated with a significant (*p* < 0.001) reduction in WAT *NNMT* expression. Bariatric surgery was also associated with a significant decrease in plasma MNA.

**Conclusions/interpretation:**

We demonstrate that WAT *NNMT* expression is regulated in human insulin resistance and type 2 diabetes and that plasma MNA correlates with increased tissue *NNMT* expression and the degree of insulin resistance, making it a potential biomarker for loss of insulin sensitivity.

**Electronic supplementary material:**

The online version of this article (doi:10.1007/s00125-014-3490-7) contains peer-reviewed but unedited supplementary material, which is available to authorised users.

## Introduction

Increased abdominal adiposity, especially expansion of visceral adipose tissue, has been identified as a major risk factor for the development of insulin resistance, type 2 diabetes and cardiovascular disease [1–3]. How visceral adiposity contributes to the development of type 2 diabetes is not fully understood. Mechanisms that have been proposed include macrophage deposition and activation, and release of proinflammatory cytokines and NEFA that exacerbate systemic insulin resistance and loss of beta cell function (reviewed in [3, 4]).

Nicotinamide, the amide form of niacin or vitamin B_3_, is a precursor in the synthesis of NAD^+^ and NADP^+^, which play a role in numerous biological processes like energy production, regulation of cellular redox state and circadian rhythm and longevity. Prolonged high intake of niacin or nicotinamide has been reported to reduce insulin sensitivity in individuals with normal or impaired glucose tolerance [5–7]. For example, individuals at risk of type 1 diabetes who were treated with nicotinamide at a dose of 2 g per day for 2 weeks showed a significant decrease in systemic insulin sensitivity, which was reversible after treatment stopped [7]. Recently, it was observed in the HPS2-THRIVE study (Heart Protection Study 2–Treatment of HDL to Reduce the Incidence of Vascular Events) that, in patients with cardiovascular disease, chronic treatment with a combination of extended-release niacin and laropiprant led to a significant increase in new-onset diabetes and disturbed glucose control in patients with established type 2 diabetes at baseline [8]. However, the mechanisms linking nicotinamide metabolism to insulin resistance are unknown. Here we explore the hypothesis that elevated nicotinamide-*N*-methyltransferase (NNMT) expression and activity may play a role in the development of human insulin resistance.

NNMT is the major nicotinamide-metabolising enzyme and catalyses the transfer of a methyl group from *S*-adenosylmethionine (SAM) to the ring nitrogen of nicotinamide thus yielding 1-methylnicotinamide (MNA) and *S*-adenosylhomocysteine (SAH). NNMT is primarily expressed in the liver but is also expressed in other organs including lung, muscle, kidney, heart, skin and adipose tissue. In adipose tissue, an increase in NNMT catalytic activity was observed in mice fed a high-fat diet for 3 months [9] whereas no parallel change in liver NNMT activity was detected [9]. *NNMT* expression both in white adipose tissue (WAT) and in liver was recently found to be upregulated in mouse models of obesity and diabetes [10]. Of note, reduction in liver and WAT *NNMT* expression by treatment with an antisense oligonucleotide was shown to protect against the development of diet-induced obesity and insulin resistance. Moreover, adipose tissue *NNMT* expression was found to be higher in obesity-prone vs obesity-resistant mouse strains [10].

Importantly, higher urinary concentrations of the NNMT product MNA and its oxidation product *N*-methyl-2-pyridone-5-carboxamide were observed in mouse and rat models of type 2 diabetes and in humans with type 2 diabetes [11]. However, it is still unclear whether plasma MNA concentrations are associated with adipose tissue *NNMT* expression, impaired insulin sensitivity or type 2 diabetes in humans. In this context we wondered whether MNA may represent a circulating biomarker of insulin resistance. We therefore investigated circulating MNA and adipose tissue *NNMT* expression in individuals with either normal glucose metabolism or insulin resistance and type 2 diabetes in three independent cohorts: (1) a cross-sectional cohort of 199 individuals undergoing abdominal surgery; (2) an exercise-intervention cohort of 60 individuals on a 3-month exercise programme and (3) a bariatric surgery intervention cohort including 55 individuals on a two-step bariatric surgery programme. In the two intervention cohorts, we assessed whether improved glucose homeostasis and/or reduced body weight are associated with changes in adipose tissue *NNMT* expression and plasma MNA concentration.

## Methods

### Study population

We included three different cohorts with a total number of 314 individuals in our study of MNA serum concentration and *NNMT* adipose tissue mRNA expression.

Individuals in all cohorts fulfilled the following inclusion criteria: (1) absence of any acute or chronic inflammatory disease as determined by a leucocyte count >8,000 × 10^9^/l, C-reactive protein (CrP) >952 nmol/l or clinical signs of infection; (2) undetectable antibodies against GAD; (3) systolic blood pressure <140 mmHg and diastolic blood pressure <90 mmHg; (4) no clinical evidence of either cardiovascular or peripheral artery disease; (5) no thyroid dysfunction; (6) no alcohol or drug abuse; (7) no pregnancy.

All study protocols have been approved by the ethics committee of the University of Leipzig (Reg. no. 031-2006 and 017-12-23012012). All participants gave written informed consent before taking part in the study.

#### Cross-sectional cohort

In the cross-sectional cohort (*n* = 199; 128 women, 71 men), we investigated MNA plasma concentrations and *NNMT* mRNA expression in paired samples of subcutaneous and omental adipose tissue in relation to measures of obesity and glucose metabolism. Individuals involved in these analyses underwent abdominal surgery for weight reduction, cholecystectomy or explorative laparoscopy between 2009 and 2012. Indications for an exploratory laparoscopy included accidents with a blunt abdominal trauma, diagnosis of endometriosis and treatment of intra-abdominal adhesions. Only adipose tissue samples were included from patients in whom laparoscopy did not identify abdominal injuries, inflammation or endometriosis. All participants had a stable weight, defined as the absence of fluctuations of >10% of body weight for at least 3 months before surgery. Adipose tissue was immediately frozen in liquid nitrogen after explantation. Histological analyses and measurement of macrophage count in adipose tissue was performed as previously described [12].

#### Exercise-intervention cohort

In a second, interventional study, we investigated adipose tissue *NNMT* expression in response to a 12-week intensive exercise intervention in 60 individuals with different degrees of glucose tolerance as previously described [13]. Individuals were divided into groups of normal glucose tolerance (NGT, *n* = 20; 9 men, 11 women), impaired glucose tolerance (IGT, *n* = 20; 9 men, 11 women) and type 2 diabetes (*n* = 20; 11 men, 9 women) on the basis of a 75 g OGTT according to the ADA criteria as previously described [14]. For details, see electronic supplementary material (ESM) Methods.

#### Bariatric surgery cohort

In addition, we measured adipose tissue *NNMT* mRNA expression and circulating MNA levels at two bariatric surgery interventions. From 55 white obese patients (38 women, 17 men), plasma samples, omental and subcutaneous adipose tissue biopsies were obtained in the context of a two-step bariatric surgery strategy with gastric sleeve resection as the first step and a Roux-en-Y gastric bypass as second step 12 ± 2 months later. The characteristics of the study population have been described previously [14].

### Measurement of body fat content, glucose metabolism, insulin sensitivity

BMI was calculated as weight divided by squared height. Hip circumference was measured over the buttocks; waist circumference was measured at the midpoint between the lower ribs and iliac crest. Percentage of body fat was measured by dual x-ray absorptiometry. Abdominal visceral and subcutaneous fat areas were calculated using MRI or computed tomography scans at the level of L4–L5 in the cross-sectional study of adipose tissue donors. Three days before the OGTT, patients documented a high-carbohydrate diet in diet protocols. The OGTT was performed after an overnight fast with 75 g standardised glucose solution (Glucodex Solution 75 g; Merieux, Montreal, QC, Canada). Venous blood samples were taken at 0, 60 and 120 min for measurement of plasma glucose concentration. Insulin sensitivity was assessed in a subgroup of 85 adipose-tissue donors including 52 individuals without type 2 diabetes and 33 patients with type 2 diabetes (Table 1) and in all participants of the intervention studies using the HOMA-IR index or the euglycaemic–hyperinsulinaemic clamp method as described [15]. Individuals with a glucose infusion rate (GIR) lower than 50 μmol kg^−1^ min^−1^ were classified as insulin resistant [15].

**Table 1:** Anthropometric and clinical characteristics of individuals who underwent abdominal surgery (cross-sectional cohort)

Characteristic	No type 2 diabetes (*n* = 111)	Type 2 diabetes (*n* = 88)	*p* value^a^
Age, years	56 ± 18	53 ± 13	0.13
Female sex, *n* (%)	73 (66)	55 (63)	0.63
Body weight, kg	95 ± 41	144 ± 41	<0.001
BMI, kg/m^2^	33 ± 13	49 ± 13	<0.001
FPG^b^, mmol/l	5.5 ± 0.9	7.3 ± 2.5	<0.001
FPI^c^, pmol/l	79 ± 108	247 ± 198	0.001
HbA_1c_^d^, % (mmol/mol)	5.4 ± 0.4 (35.5 ± 4.4)	6.6 ± 1.1 (48.6 ± 12.1)	<0.001
GIR^e^, μmol kg^−1^ min^−1^	87 ± 28	35 ± 24	<0.001
TG^f^, mmol/l	1.2 ± 0.5	2.0 ± 1.0	<0.001
LDL-C^g^, mmol/l	3.1 ± 1.0	2.7 ± 0.8	0.04
HDL-C^g^, mmol/l	1.4 ± 0.5	1.2 ± 0.4	0.01

Data are given as mean ± SD for parametric variables or number (column percentage) for categorical variables

^a^The *p* values were determined using Student’s *t* test for continuous parametric values and *χ* ^2^ tests for categorical values

^b^Fasting plasma glucose, *n* = 175 (95 no T2D, 80 T2D)

^c^Fasting plasma insulin, *n* = 120 (58 no T2D, 62 T2D)

^d^HbA_1c_, *n* = 110 (53 no T2D, 57 T2D)

^e^Glucose infusion rate, *n* = 85 (52 no T2D, 33 T2D)

^f^Triacylglycerols, *n* = 119 (52 no T2D, 67 T2D)

^g^LDL-cholesterol, HDL-cholesterol, *n* = 116 (51 no T2D, 65 T2D)

T2D, type 2 diabetes

### Analyses of blood samples

All baseline blood samples were collected between 08:00 hours and 10:00 hours after an overnight fast. Plasma insulin was measured with an enzyme immunometric assay for the IMMULITE automated analyser (Diagnostic Products Corporation, Los Angeles, CA, USA). Serum lipid profiles were measured as previously described [16].

### *NNMT* mRNA expression studies

Human *NNMT* mRNA expression was measured by quantitative real-time RT-PCR in a fluorescent temperature cycler using the TaqMan assay and fluorescence was detected on an ABI PRISM 7000 sequence detector (Applied Biosystems, Darmstadt, Germany). For details, see ESM Methods.

### Quantification of MNA concentrations in plasma

Plasma MNA was measured by liquid chromatography with tandem mass spectrometry (LCMSMS) using an electrospray ionisation–triple quadrupole mass spectrometer coupled to a liquid chromatography system. See ESM Methods for further details.

### Statistical analysis

The homoscedastic two-sided Student’s *t* test was used to compare clinical characteristics between different groups of individuals (e.g. *NNMT* expression between non-diabetic and diabetic individuals). The paired *t* test was used to compare clinical variables within individual participants (e.g. adipose tissue *NNMT* expression before and after exercise intervention or before and after bariatric surgery). A *p* value < 0.05 was considered to be statistically significant.

Spearman’s correlation coefficients were calculated to evaluate the relationships between different clinical characteristics among the same group of individuals (e.g. to investigate the association between plasma MNA concentrations and adipose tissue *NNMT* expression). For the latter, data were log_10_-transformed to achieve normal distribution.

Linear regression modelling was performed using SAS ProcGLM (SAS version 9.2, SAS Institute, Cary, NC, USA).

## Results

### Cross-sectional cohort

Anthropometric, demographic and clinical characteristics of the study participants are summarised in Table 1. Overall, adipose tissue and plasma samples were collected from 199 individuals undergoing abdominal surgery, 88 of them having been diagnosed with type 2 diabetes before surgery. Of the 88 patients with type 2 diabetes, 56 were treated by diet and exercise without any pharmacotherapy, 17 received metformin, 20 were treated with insulin (with or without metformin), one received glimepiride and one pioglitazone.

**Fig. 1:**
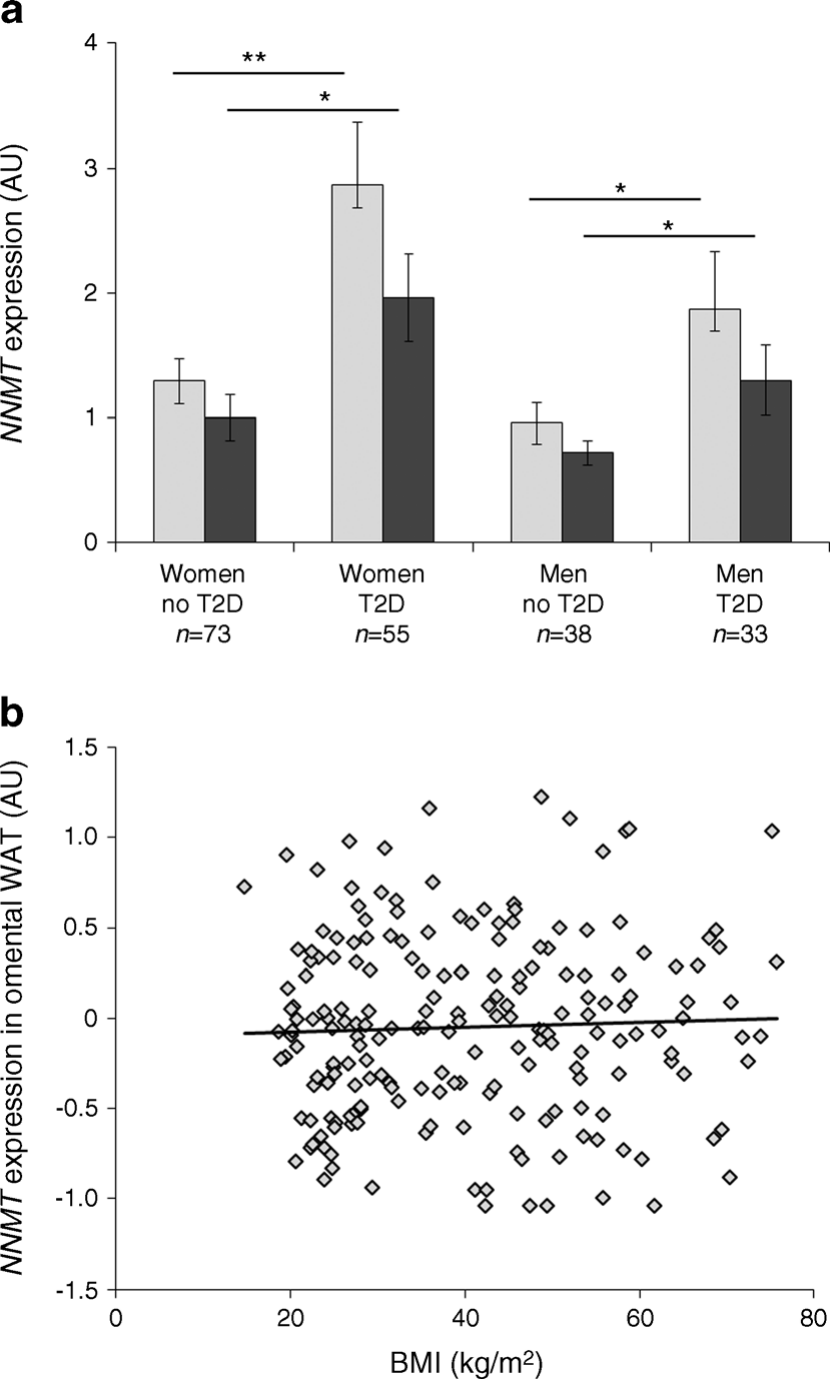
Differences in WAT *NNMT* expression with type 2 diabetes and BMI. (**a**) Expression of *NNMT* in either omental (light-grey bars) or subcutaneous (dark-grey bars) adipose tissue was significantly higher in individuals with type 2 diabetes compared with non-diabetic individuals. (**b**) There was no correlation between omental WAT *NNMT* expression and BMI (*r* ^2^ = 0.001). AU, arbitrary units; T2D, type 2 diabetes. **p < 0.01, *p < 0.05 for indicated comparisons

Figure 1a shows the difference in adipose tissue *NNMT* expression between individuals with type 2 diabetes and those without type 2 diabetes. In both subcutaneous and omental WAT, *NNMT* expression was significantly (approximately twofold) higher in the diabetic patients than in the non-diabetic patients, irrespective of sex. Under the assumption of a log-normal distribution of *NNMT* expression, the ratio of the geometric means of *NNMT* expression between patients with type 2 diabetes and non-diabetic patients was 1.82 (95% CI 1.22, 2.72) for omental adipose tissue in women, 1.54 (0.91, 2.44) for omental adipose tissue in men, 1.67 (1.16, 2.41) for subcutaneous adipose tissue in women and 1.52 (0.95, 2.44) for subcutaneous adipose tissue in men.

In contrast, no significant association between BMI and adipose tissue *NNMT* expression was observed. There was no correlation between *NNMT* expression in omental (Fig. 1b) or subcutaneous WAT (not shown) and BMI. There was a trend across BMI tertiles towards an increase in omental WAT *NNMT* expression that was, however, not statistically significant (*p* = 0.2)

**Fig. 2:**
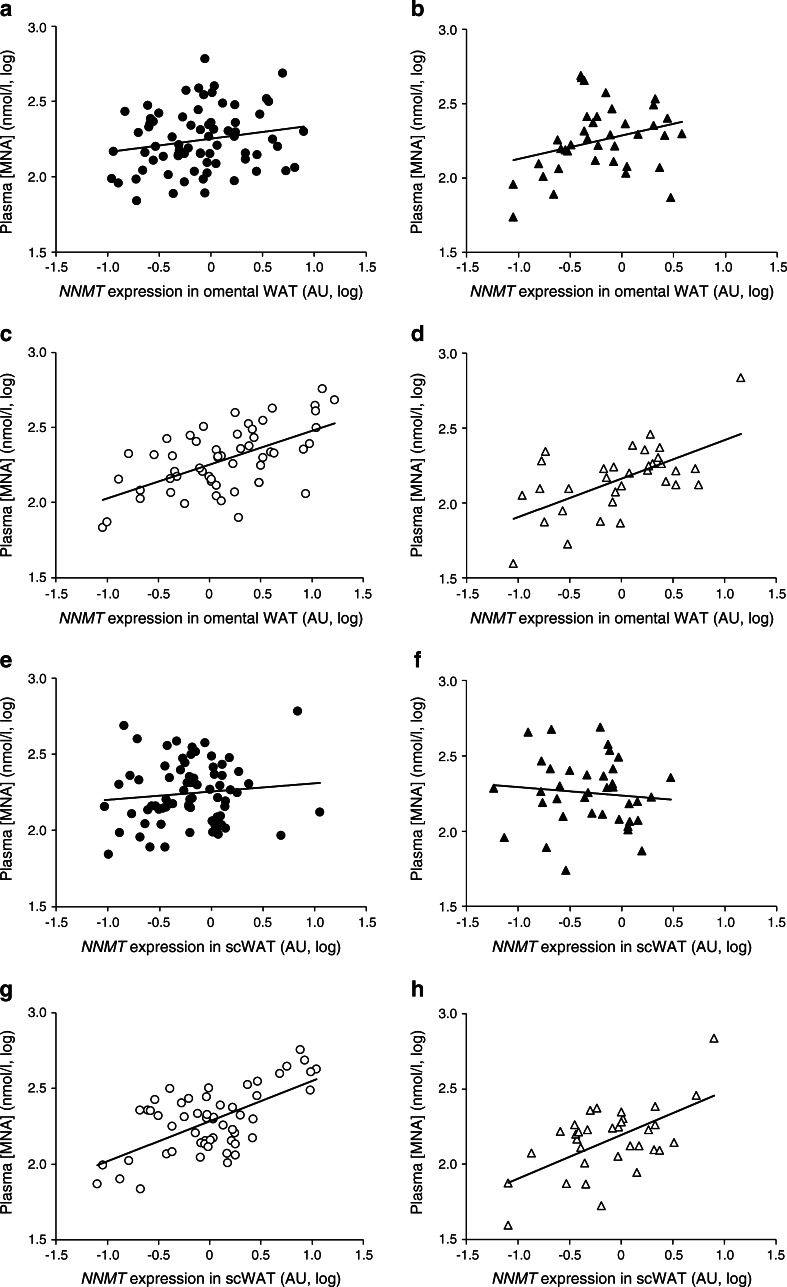
Correlation between plasma MNA concentration and *NNMT* expression in omental (**a**–**d**) and subcutaneous (**e**–**h**) WAT. Circles, women; triangles, men; black symbols, non-diabetic individuals; white symbols, individuals with type 2 diabetes. Data were log_10_-transformed to achieve normal distribution Correlation coefficients were as follows: (**a**) *r* ^2^ = 0.04, NS; (**b**) *r* ^2^ = 0.09, NS; (**c**) *r* ^2^ = 0.35, *p* < 0.001; (**d**) *r* ^2^ = 0.37, *p* < 0.001; (**e**) *r* ^2^ = 0.01, NS; (**f**) *r* ^2^ = 0.01, NS; (**g**) *r* ^2^ = 0.39, *p* < 0.001; (**h**) *r* ^2^ = 0.35, *p* < 0.001. AU, arbitrary units; scWAT, subcutaneous WAT

Interestingly, plasma MNA concentrations did not correlate with either omental (Fig. 2a, b) or subcutaneous (Fig. 2e, f) adipose tissue *NNMT* expression in non-diabetic individuals. However, there was a statistically significant positive association (*r* = 0.6, *p* < 0.001) between plasma MNA concentration and adipose tissue *NNMT* expression in both adipose depots and for both women and men with type 2 diabetes (Fig. 2c, d and g, h). In a linear regression analysis with *NNMT* expression as independent and MNA as dependent variable (both log_10_-transformed), there was a significant interaction of the regression parameter with diabetes status (*p* = 0.015 for omental, *p* < 0.001 for subcutaneous WAT) that remained significant after adjustment for age, BMI and sex (*p* = 0.016 for omental, *p* < 0.001 for subcutaneous WAT).

Of note, although there was no significant association of adipose tissue *NNMT* expression with BMI (Fig. 1b), MNA concentrations significantly correlated with omental or subcutaneous adipose tissue *NNMT* expression in all individuals with a BMI above the median BMI of 39 kg/m^2^ (*r* = 0.56 for omental and *r* = 0.5 for subcutaneous adipose tissue *NNMT*, *p* < 0.001 each). In contrast, in individuals with a BMI below median we only found a significant correlation between circulating MNA and omental *NMMT* expression (*r* = 0.24, *p* = 0.02), but no significant correlation for subcutaneous (*r* = 0.15, *p* = 0.15) adipose tissue (ESM Fig. 1).

**Fig. 3:**
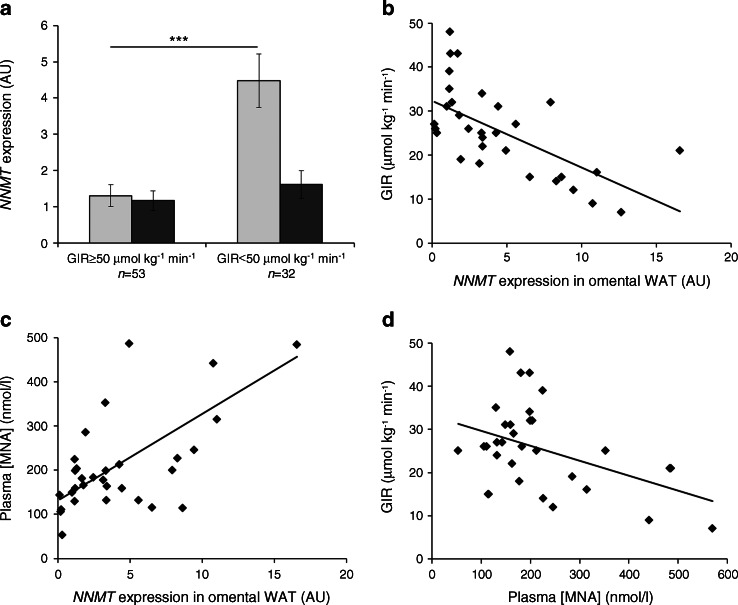
Association of adipose tissue *NNMT* expression and insulin resistance for the subgroup of patients for whom there were clamp data available (*n* = 85). (**a**) Expression of *NNMT* in omental (light-grey bars) but not subcutaneous (dark-grey bars) adipose tissue was significantly higher in individuals with GIR <50 μmol kg^−1^ min^−1^ (***p < 0.001 vs GIR ≥50 μmol kg^−1^ min^−1^). (**b**) Negative correlation of tissue *NNMT* expression with insulin sensitivity in insulin-resistant individuals (GIR <50 μmol kg^−1^ min^−1^, *n* = 32, *r* ^2^ = 0.41, *p* < 0.001). (**c**) Positive correlation between *NNMT* expression in omental adipose tissue and plasma MNA concentrations in insulin-resistant individuals (*r* ^2^ = 0.44, *p* < 0.001). (**d**) Negative correlation of GIR and plasma MNA in insulin-resistant individuals (*r* ^2^ = 0.19, *p* < 0.05). AU, arbitrary units

In individuals for whom hyperinsulinaemic–euglycaemic clamp data were available (*n* = 85), *NNMT* expression in omental adipose tissue was about 3.5-fold higher in insulin-resistant individuals (*n* = 32, *p* < 0.001) than in insulin-sensitive individuals (*n* = 53) (Fig. 3a). Insulin resistance was defined by a GIR lower than 50 μmol kg^−1^ min^−1^ [15]. In contrast, the difference in subcutaneous adipose tissue *NNMT* mRNA between insulin-resistant and insulin-sensitive individuals did not reach statistical significance (*p* = 0.3). In insulin-resistant individuals, we found significant correlations between GIR and *NNMT* expression in omental (Fig. 3b, *r* = 0.64, *p* < 0.001) and subcutaneous adipose tissue (*r* = 0.4, *p* = 0.02, ESM Fig. 2). As there was also a positive association between plasma MNA concentration and adipose tissue *NNMT* expression in this study group (Fig. 3c, *r* = 0.66, *p* < 0.001), plasma MNA levels significantly correlate with the degree of insulin resistance (Fig. 3d, *r* = 0.44, *p* = 0.01). These correlations were not observed in insulin-sensitive individuals.

### Exercise-intervention cohort

**Fig. 4:**
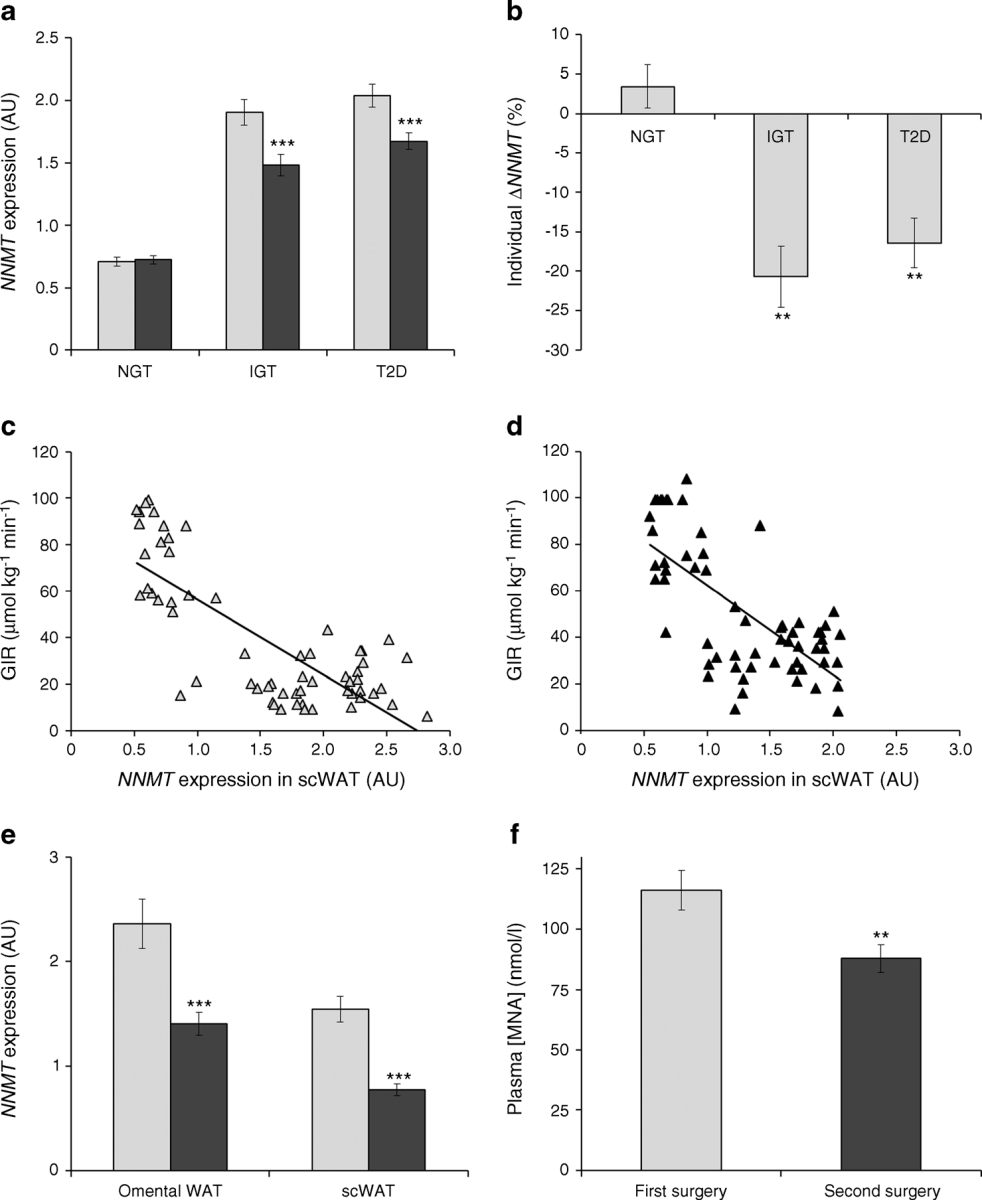
Interventions that improve insulin sensitivity are associated with reduced adipose tissue *NNMT* expression and plasma MNA concentrations. (**a**) *NNMT* expression in subcutaneous adipose tissue before (light-grey bars) and after exercise intervention (dark-grey bars) for individuals with NGT, IGT or type 2 diabetes (*n* = 20 each; ***p < 0.001 vs pre-exercise, paired *t* test). (**b**) Intra-individual change in *NNMT* expression (percentage of pre-exercise expression, **p < 0.001 vs NGT, paired *t* test) upon exercise. (**c**) Correlation between adipose tissue *NNMT* expression and GIR during hyperinsulinaemic–euglycaemic clamp before the exercise programme (*r* ^2^ = 0.61, *p* < 0.001) and (**d**) after the exercise programme (*r* ^2^ = 0.50, *p* < 0.001). (**e**) Omental and subcutaneous WAT *NNMT* expression in the context of a two-step bariatric surgery (*n* = 45; ***p < 0.001 vs first surgery, paired *t* test). (**f**) Change in plasma MNA concentration upon two-step bariatric surgery (*n* = 38; **p = 0.001 vs first surgery, paired *t* test). Light-grey bars, samples obtained at first surgery; dark-grey bars, samples obtained at second surgery 12 ± 2 months later. AU, arbitrary units; scWAT, subcutaneous WAT; T2D, type 2 diabetes

The association between adipose tissue *NNMT* expression and insulin resistance observed in the cross-sectional cohort could be confirmed in an independent cohort (*n* = 60, 20 each of individuals with NGT, IGT and type 2 diabetes) of individuals that participated in a 12-week exercise-intervention programme consisting of three 1 h sessions of exercise per week. Clinical and anthropometric characteristics of study participants before and after exercise are summarised in Table 2. In all three groups—NGT, IGT and type 2 diabetes—the physical training programme led to a significant reduction in body weight and an improvement in glucose metabolism. Subcutaneous adipose tissue *NNMT* expression was significantly higher in patients with type 2 diabetes (2.9-fold, *p* < 0.001) or IGT (2.7-fold, *p* < 0.001) than in individuals with NGT (Fig. 4a). Importantly, endurance training led to a significant decrease in adipose tissue *NNMT* expression in individuals with IGT (−21%, *p* < 0.001) and in those with type 2 diabetes (−16%, *p* < 0.001) but not in NGT individuals (+3%, *p* = 0.4, Fig. 4b). There was a strong correlation between GIR and adipose tissue *NNMT* expression both before (*r* = 0.78, *p* < 0.001) and after the exercise programme (*r* = 0.70, *p* < 0.001) (Fig. 4c, d).

**Table 2:** Anthropometric and clinical characteristics of the exercise-intervention cohort at baseline and after the 12-week exercise programme

Characteristic	NGT (*n* = 20)		IGT (*n* = 20)		Type 2 diabetes (*n* = 20)	
	Baseline	After exercise	Baseline	After exercise	Baseline	After exercise
Age, years	33 ± 11		56 ± 12***		53 ± 7***	
Female sex, *n* (%)	11 (55)		11 (55)		9 (45)	
Weight, kg	69.6 ± 14.4	68.2 ± 13.7^‡‡^	87.6 ± 16.4***	84.5 ± 16.2^‡‡‡^	94.7 ± 19.7***	93.0 ± 18.2^‡‡‡^
BMI, kg/m^2^	23.3 ± 3.2	22.8 ± 3.1^‡‡^	29.6 ± 6.0***	28.5 ± 5.9^‡‡‡^	32.2 ± 6.0***	31.7 ± 5.5^‡^
Body fat, %	24.5 ± 3.2	23.3 ± 2.7^‡‡‡^	34.9 ± 8.3***	31.5 ± 7.5^‡‡‡^	38.2 ± 8.0***	35.2 ± 7.7^‡‡‡^
FPG, mmol/l	5.2 ± 0.5	5.0 ± 0.4	5.7 ± 0.6**	5.5 ± 0.6	6.2 ± 0.6***^,††^	5.8 ± 0.5^‡‡^
2 h glucose, mmol/l	6.0 ± 0.8	5.6 ± 0.6^‡‡^	9.4 ± 0.9***	8.1 ± 1.4^‡‡‡^	13.1 ± 1.5***^,†††^	12.6 ± 2.5
Basal insulin, pmol/l	66 ± 35	58 ± 27^‡^	695 ± 493***	379 ± 324^‡‡‡^	319 ± 212***^,††^	234 ± 120^‡‡^
Whole-body glucose uptake, μmol kg^−1^ min^−1^	76 ± 17	85 ± 15^‡‡‡^	19 ± 9***	36 ± 16^‡‡‡^	21 ± 9***	32 ± 11^‡‡‡^

Data are given as mean ± SD for parametric variables or number (column percentage) for categorical variables

**p < 0.01 and ***p < 0.001 vs NGT (*t* test)

^††^ *p* < 0.01 and ^†††^ *p* < 0.001 vs IGT (*t* test)

^‡^ *p* < 0.05, ^‡‡^ *p* < 0.01 and ^‡‡‡^ *p* < 0.001 vs before exercise (paired *t* test)

### Bariatric surgery cohort

In a third group of patients who were morbidly obese (*n* = 55), *NNMT* expression in subcutaneous and omental adipose tissue and plasma MNA concentrations were analysed in the context of a two-step bariatric surgery procedure at the first-step surgery (sleeve gastrectomy) and the second-step surgery (gastric bypass) 12 ± 2 months after the baseline surgery. Overall anthropometric and clinical characteristics of these patients before first and second surgery are summarised in Table 3. Sleeve gastrectomy was associated with a significant reduction in body weight and improvement in glucose homeostasis. There was a significant reduction in *NNMT* expression post-surgery in both subcutaneous (−50%, *p* < 0.001 vs first surgery) and omental (−41%, *p* < 0.001 vs first surgery) adipose tissue (Fig. 4e). This reduction in expression was reflected by a significant reduction in plasma MNA concentration (−24%, *p* = 0.001 vs first surgery, Fig. 4f).

**Table 3:** Anthropometric and clinical characteristics of the bariatric surgery cohort (*n* = 55)

Characteristic	First-step surgery	Second-step surgery	*p* value^a^
Age, years	44.5 ± 11.0		
Female sex, *n* (%)	38 (69)		
Type 2 diabetes, *n* (%)	31 (56)	4 (7)	<0.001
Weight, kg	158 ± 30	108 ± 25	<0.001
BMI, kg/m^2^	53.6 ± 7.3	36.7 ± 6.8	<0.001
HbA_1c_, % (mmol/mol)	6.5 ± 1.0 (47.5 ± 11.0)	5.4 ± 0.7 (35.4 ± 7.8)	<0.001
FPG, mmol/l (no T2D)	5.6 ± 0.4	5.3 ± 0.4	0.06
FPG, mmol/l (T2D)	7.2 ± 1.5	5.8 ± 1.5	<0.001
FPI, pmol/l	275 ± 239	93 ± 80	<0.001
TG, mmol/l	2.2 ± 1.0	1.5 ± 0.7	<0.001

Data are given as mean ± SD for parametric variables or number (column percentage) for categorical variables

Morbidly obese individuals underwent a two-step bariatric surgery intervention with sleeve gastrectomy as a first-step surgery and a Roux-en-Y gastric bypass surgery 12 ± 2 months after the first-step surgery

^a^The *p* values were determined using paired Student’s *t* test for continuous parametric values and *χ* ^2^ tests for categorical values

T2D, type 2 diabetes; TG, triacylglycerol

## Discussion

This study demonstrates for the first time that *NNMT* mRNA in adipose tissue and circulating levels of the product of NNMT, MNA, were higher in humans with insulin resistance or type 2 diabetes and correlated with the extent of insulin resistance. An additional novel finding is that interventions improving insulin sensitivity—exercise and bariatric surgery—are associated with a decrease in adipose tissue *NNMT* expression and, for bariatric surgery, a reduction in plasma MNA levels.

Previously, higher plasma MNA levels had been observed in patients with type 2 diabetes compared with healthy controls 5 h after a 100 mg nicotinamide challenge [17]. Neither *NNMT* expression nor activity was determined in that study and higher plasma MNA concentrations were attributed to slower MNA clearance. In addition, there was no significant difference in MNA plasma concentration between the diabetic individuals and normal controls before the nicotinamide challenge. However, the study was comparatively small (*N* = 28), and no details were given regarding body weight or insulin resistance in either group of patients. Notably, prolonged treatment (2 weeks) with nicotinic acid or nicotinamide has been demonstrated to cause insulin resistance in individuals with NGT or IGT [5–7]. Although no MNA plasma concentrations were measured in these clinical studies, experiments in rats demonstrated that nicotinamide administration results in a marked increase in plasma MNA concentration [17, 18], indicating that a similar rise in plasma MNA can be expected in humans. Interestingly, administration of either nicotinamide or MNA to healthy rats had very similar effects on glucose homeostasis, leading to an increase in both blood glucose and insulin concentrations. This suggests that the loss of insulin sensitivity upon nicotinic acid or nicotinamide treatment could be mediated by MNA.

Higher urinary MNA concentrations were observed in patients with type 2 diabetes compared with healthy controls and also in *db*/*db* mice and obese Zucker rats [11] and mice on high-fat diet [19].

Importantly, we observed a correlation between plasma MNA levels and adipose tissue *NNMT* expression only in individuals with type 2 diabetes or high BMI but not in those without diabetes or who had lower BMI (Fig. 2 and ESM Fig. 1). Although the underlying mechanisms for this observation are not clear, we postulate that individuals without diabetes may be able to mitigate the influence of high WAT *NNMT* expression on plasma MNA levels by a mechanism that is compromised in patients with diabetes. Alternatively, the contribution of WAT *NNMT* to circulating MNA concentrations relative to other organs, especially liver, may be higher in insulin-resistant or diabetic individuals. This would be in line with a report by Riederer et al [9] demonstrating that high-fat feeding of mice leads to a selective increase in WAT NNMT activity whereas activity in the liver remains unchanged. Moreover, mice strains known to be very susceptible to developing diet-induced obesity were found to have high WAT *NNMT* expression whereas WAT *NNMT* expression was low in obesity-resistant strains. No such relationship was observed between liver NNMT expression and susceptibility to diet-induced obesity [10]. Thus, our data may suggest that in individuals with type 2 diabetes or high BMI, WAT *NNMT* makes a larger contribution to circulating MNA levels—leading to the observed correlation between systemic MNA and adipose tissue *NNMT* expression–than in individuals without diabetes and with a lower BMI. In healthy lean individuals, liver NNMT activity may predominantly determine circulating MNA levels. Kraus et al [10] described higher NNMT protein levels in both WAT and liver in mouse models of type 2 diabetes or diet-induced obesity, though they did not measure NNMT activity. We cannot exclude the possibility that increased NNMT activity in the liver of patients with type 2 diabetes and obesity may, in addition to increased expression in WAT, also contribute to the observed associations in these patients. A limitation of our study is therefore that hepatic NNMT expression and activity could not be determined, because liver biopsies were not available for these cohorts.

The correlation between adipose tissue *NNMT* expression and insulin resistance—the latter being defined by a low GIR in the hyperinsulinaemic–euglycaemic clamp—was confirmed in two independent cohorts (see Figs 3b, 4c, d). On the other hand, the association of plasma MNA levels with GIR was less robust (Fig. 3d) and was only observed in insulin-resistant individuals. Therefore the value of plasma MNA as a diagnostic biomarker for insulin resistance is still limited. The cohorts analysed in our study represent a diverse clinical spectrum of obesity and insulin resistance over a broad age range. Although this disease spectrum reflects clinical reality, it may be associated with confounding factors unrelated to insulin sensitivity that could influence circulating MNA levels. Another potential limitation of our study includes the assessment of total-body fat mass and adipose tissue distribution using dual-energy x-ray absorptiometry (DEXA), MRI or computerised tomography scans at only one defined abdominal segment. We can therefore not rule out the possibility that using whole-body MRI scans may identify additional correlations of circulating MNA and/or *NNMT* expression data with total-body fat mass and fat distribution. Thus, further clinical studies, preferably with more sophisticated measurements, in cohorts carefully matched by age, BMI and disease, will be required to fully explore the usefulness of MNA as a biomarker for insulin resistance and to assess its value beyond existing markers (e.g. high-sensitivity C-reactive protein or HbA_1c_). Yet, our data already demonstrate that in individuals with type 2 diabetes or insulin resistance, a high plasma MNA level indicates a high level of tissue *NNMT* expression and that MNA could be used to identify patients eligible for therapy with an NNMT inhibitor and to monitor their treatment response.

The mechanisms by which increased NNMT activity and higher MNA levels could influence insulin sensitivity are unclear. Potential pathways may involve the generation of reactive oxygen species [17, 20, 21], an effect on cellular NAD^+^ levels or sirtuin activity [10, 21–23] or a decrease in cellular methylation potential (e.g. via lowering the SAM/SAH ratio) and resulting changes in protein and DNA methylation [10, 18, 24]. For the latter, it is notable that *NNMT* is overexpressed in various forms of human cancer including lung, kidney, bladder and colorectal cancer [25–28] and oral squamous cell carcinoma [29]. It has been suggested that MNA could provide a sink for methyl groups and that the resulting protein hypomethylation may promote oncogenesis.

In summary, we have demonstrated that adipose tissue *NNMT* expression is increased in insulin resistance and type 2 diabetes and correlates with the severity of insulin resistance. Moreover, in insulin-resistant individuals, plasma MNA is a biomarker for adipose tissue *NNMT* expression and the extent of systemic insulin resistance. Thus, NNMT inhibition may provide a novel therapeutic approach for insulin resistance. The molecular mechanism by which NNMT could regulate insulin sensitivity is yet unknown and various possibilities are currently being investigated.

## Electronic supplementary material

Below is the link to the electronic supplementary material.

ESM Methods (PDF 26 kb)

ESM Fig. 1 (PDF 103 kb)

ESM Fig. 2 (PDF 40 kb)

## Abbreviations

CRP: C-reactive protein
GIR: Glucose infusion rate
IGT: Impaired glucose tolerance
MNA: 1-Methylnicotinamide
NGT: Normal glucose tolerance
NNMT: Nicotinamide-*N*-methyltransferase
SAH: *S*-Adenosylhomocysteine
SAM: *S*-Adenosylmethionine
WAT: White adipose tissue
