# Supplementary material for: Association of nicotinamide-N-methyltransferase mRNA expression in human adipose tissue and the plasma concentration of its product, 1-methylnicotinamide, with insulin resistance
Source: Diabetologia. 2015 Jan 18;58(4):799–808. doi: 10.1007/s00125-014-3490-7 (PMC4351435; doi:10.1007/s00125-014-3490-7)
Supplement: Supplementary file 1 — (PDF 26 kb) [file 125_2014_3490_MOESM1_ESM.pdf]

Electronic supplementary material (ESM)

ESM methods

Exercise intervention

Sixty individuals (N=20 with normal glucose tolerance (NGT), N=20 with impaired glucose tolerance (IGT), N=20 with type-2 diabetes) were enrolled in a 12 weeks supervised physical training program. NGT was defined by a fasting plasma glucose <6.0 mmol/l, and a 120-min plasma glucose <7.8 mmol/l. IGT was defined by a fasting plasma glucose <6.0 mmol/l, a 120-min plasma glucose > 7.8 mmol/l and <11.1 mmol/l. Type-2 diabetes was defined by a fasting plasma glucose > 7.0 mmol/l and/or a 120min OGTT glucose >11.1 mmol/l. Study participants performed exercise at 3 days per week. Each training session lasted in total 60 minutes and included 10 minutes of warming up, 20 minutes of biking or running followed by 20 minutes of swimming and 10 minutes of cooling down. All participants completed a graded bicycle-ergometer test to volitional exhaustion and had maximal oxygen uptake measured with an automated open circuit gas analysis system at baseline, after 4 and 12 weeks of training. The highest oxygen uptake/minute reached was defined as the maximal oxygen uptake, and subjects subsequently trained at their individual submaximal heart rate defined as 70-80 % of the individual maximal heart rate during the bicycle-ergometer test. At baseline, after 4 and 12 weeks of training (48 hours after the last training session), blood samples were obtained in the fasting state and measurements of anthropometric parameters were performed. Subcutaneous adipose tissue biopsies were taken before and after the exercise program.

NNMT mRNA expression studies

Samples of subcutaneous and omental adipose tissue were immediately frozen in liquid nitrogen after biopsy. Total RNA was isolated using TRIzol (Life Technologies, Grand Island, NY), and 1 µg RNA was reverse transcribed with standard reagents (Life Technologies). From each RT-PCR, 1 µl was amplified in a 20-µl PCR using the Brilliant SYBR Green QPCR Core Reagent kit from Stratagene (La Jolla, CA) according to the manufacturer’s instructions. *NNMT* mRNA expression was measured

by quantitative real-time RT-PCR in a fluorescent temperature cycler using a TaqMan® Gene Expression Assay (Hs00196287_m1) that contains premixed fluorescently labelled probes and TaqMan® 2xUniversal PCR Master Mix (Applied Biosystems, Darmstadt, Germany). Samples were incubated in the ABI PRISM 7000 sequence detector for an initial denaturation at 95°C for 10 min, followed by 40 PCR cycles, each cycle consisting of 95 °C for 15 s, 60 °C for 1 min, and 72 °C for 1 min. Fluorescence emissions were monitored after each cycle. *NNMT* mRNA expression was calculated relative to the mRNA expression of Hypoxanthine-Guanine-Phosphoribosyltransferase 1 (HPRT1), determined by a pre-developed TaqMan® Assay (Hs01003267_m1; Applied Biosystems): Normalization by endogenous control results in arbitrary units (AU).

Quantification of plasma MNA

Plasma MNA was measured by liquid chromatography with tandem mass spectrometry (LCMSMS) using an electrospray ionization-triple quadrupol mass spectrometer (Quantum Ultra,Thermo) coupled to a liquid chromatography system (UltiMate3000, Dionex) controlled by XCalibur 2.0.7 software with Dionex Chrom MS link 6.80. Chromatographic separation was achieved on a Kinetex Hilic 2.6 µm, 1x100 mm analytical column (Phenomenex) at 65 °C with a flow rate of 350 µL/min; autosampler temperature was set to 10 °C. A sample volume of 20 µL was injected onto the column. Eluents consisted of water/0.1 % formic acid (A) and acetonitrile/0.1 % formic acid (B). Initial conditions (0-0.85 min) were 98% B, then a ramp was applied within 1.1 min to 5 % B, a condition which remained until 3.25 min, before the system was equilibrated to initial conditions within 2.15 min, resulting in a total run time of 5.5 min per sample. The column flow was directly converted into the H-electrospray ionization (HESI) source of the mass spectrometer, which was operated in the positive ion mode. Capillary and vaporizer temperatures were maintained at 320 °C and 350 °C, respectively. Sheath gas and auxiliary gas were operated at 60 and 15 (pressure, arbitrary units), no ion sweep gas was applied. Collision energy for single reaction monitoring and tube lens offset were adjusted to 19 V and 70 V, respectively. Quantification was performed via peak area ratios (selected reaction monitoring m/z 137 -> m/z 94) applied to internal standard d4-N-methylnicotinamide

(selected reaction monitoring m/z 141 -> m/z 98) in an external calibration curve using XCalibur QuanBrowser.

Sample preparation started with protein precipitation by addition of 80 µL of a freshly prepared acetonitrile solution containing 0.25 µmol/l d4-MNA to 20 µL plasma sample in a 0.5 mL plastic tube. After rigorous mixing the samples were incubated at 5 °C in the refrigerator for 20 min before they were centrifuged at 16,100 rcf at 4 °C for 20 min. 60 µL of the supernatant was transferred to a fresh vial which was then tightly sealed before undergoing analysis.

60 samples per day were prepared and analyzed overnight. Each day, samples for a daily external calibration curve were prepared by using the exact acetonitrile/0.25 µmol/l d4-MNA - lot which has been used for the samples. Thus, the eight calibration samples contained 0.25 µmol/l internal standard different amounts of MNA ranging from 0 – 1000 nmol/l.
