# Supplementary material for: Association of nicotinamide-N-methyltransferase mRNA expression in human adipose tissue and the plasma concentration of its product, 1-methylnicotinamide, with insulin resistance
Source: Diabetologia. 2015 Jan 18;58(4):799–808. doi: 10.1007/s00125-014-3490-7 (PMC4351435; doi:10.1007/s00125-014-3490-7)

ESM Fig. 1. Relationship between MNA concentrations and *NNMT* expression in adipose tissue dependent on BMI for the cross-sectional cohort. Data were $\log_{10}$-transformed to achieve normal distribution. ***a***,***b***, omental adipose tissue; ***c***,***d***, subcutaneous adipose tissue. Circles: Individuals with BMI<39 (*n*=99). Triangles: Individuals with BMI>39 (*n*=99). *p<0.05, ***p<0.001.

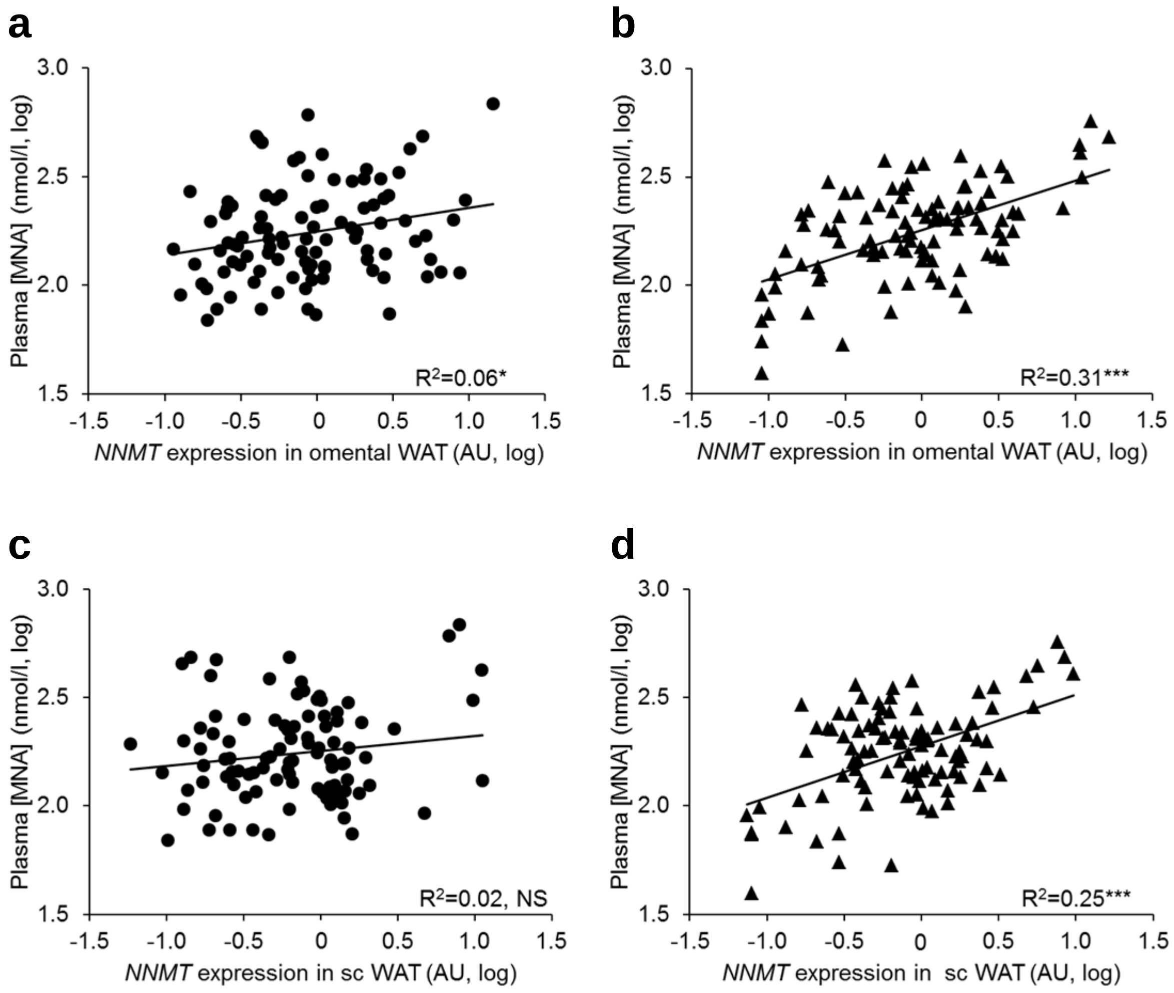

Supplement: Supplementary file 2 — (PDF 103 kb) [file 125_2014_3490_MOESM2_ESM.pdf]
