# Supplementary material for: Association of nicotinamide-N-methyltransferase mRNA expression in human adipose tissue and the plasma concentration of its product, 1-methylnicotinamide, with insulin resistance
Source: Diabetologia. 2015 Jan 18;58(4):799–808. doi: 10.1007/s00125-014-3490-7 (PMC4351435; doi:10.1007/s00125-014-3490-7)

ESM Fig. 2. Negative correlation between glucose infusion rate and *NNMT* expression in subcutaneous adipose tissue in insulin-resistant individuals (*n*=32). *p<0.05

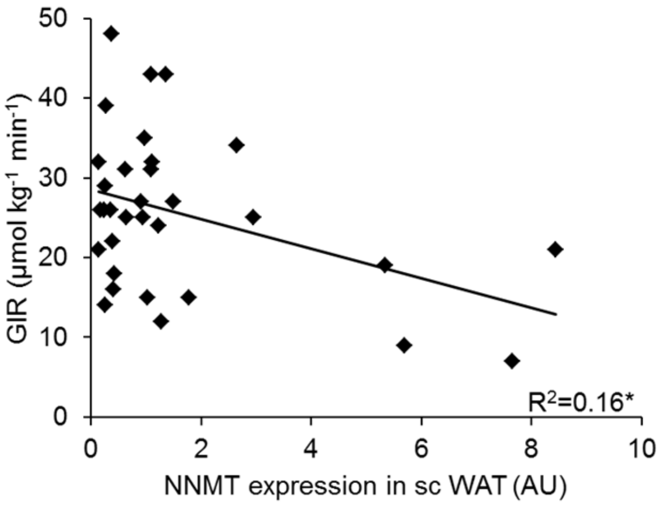

Supplement: Supplementary file 3 — (PDF 40 kb) [file 125_2014_3490_MOESM3_ESM.pdf]
